# Onconase responsive genes in human mesothelioma cells: implications for an RNA damaging therapeutic agent

**DOI:** 10.1186/1471-2407-10-34

**Published:** 2010-02-05

**Authors:** Deborah A Altomare, Susanna M Rybak, Jianming Pei, Jacob V Maizel, Mitchell Cheung, Joseph R Testa, Kuslima Shogen

**Affiliations:** 1 Women's Cancer Program, Fox Chase Cancer Center, 333 Cottman Avenue, Philadelphia, PA, 19111, USA; 2 Cancer Genetics & Signaling Program, Fox Chase Cancer Center, 333 Cottman Avenue, Philadelphia, PA, 19111, USA; 3 Alfacell Corporation, 300 Atrium Drive, Somerset, NJ, 08873, USA; 4 Current address: Burnett School of Biomedical Sciences, University of Central Florida, 6900 Lake Nona Blvd., Orlando, FL, 32827, USA

## Abstract

**Background:**

Onconase represents a new class of RNA-damaging drugs. Mechanistically, Onconase is thought to internalize, where it degrades intracellular RNAs such as tRNA and double-stranded RNA, and thereby suppresses protein synthesis. However, there may be additional or alternative mechanism(s) of action.

**Methods:**

In this study, microarray analysis was used to compare gene expression profiles in untreated human malignant mesothelioma (MM) cell lines and cells exposed to 5 μg/ml Onconase for 24 h. A total of 155 genes were found to be regulated by Onconase that were common to both epithelial and biphasic MM cell lines. Some of these genes are known to significantly affect apoptosis (IL-24, TNFAIP3), transcription (ATF3, DDIT3, MAFF, HDAC9, SNAPC1) or inflammation and the immune response (IL-6, COX-2). RT-PCR analysis of selected up- or down-regulated genes treated with varying doses and times of Onconase generally confirmed the expression array findings in four MM cell lines.

**Results:**

Onconase treatment consistently resulted in up-regulation of IL-24, previously shown to have tumor suppressive activity, as well as ATF3 and IL-6. Induction of ATF3 and the pro-apoptotic factor IL-24 by Onconase was highest in the two most responsive MM cell lines, as defined by DNA fragmentation analysis. In addition to apoptosis, gene ontology analysis indicated that pathways impacted by Onconase include MAPK signaling, cytokine-cytokine-receptor interactions, and Jak-STAT signaling.

**Conclusions:**

These results provide a broad picture of gene activity after treatment with a drug that targets small non-coding RNAs and contribute to our overall understanding of MM cell response to Onconase as a therapeutic strategy. The findings provide insights regarding mechanisms that may contribute to the efficacy of this novel drug in clinical trials of MM patients who have failed first line chemotherapy or radiation treatment.

## Background

Onconase (ranpirnase), an extremely stable endoribonuclease that was originally isolated from the oocytes of *Rana pipiens*, is part of a paradigm shift in drug development. The intracellular target is RNA, not DNA or protein. Onconase damage to tRNA [1-3] causes activation of the caspase cascade in mammalian cells and results in apoptosis [2,4]. Although Onconase cleaves tRNA at unique sites compared to other pancreatic type RNases [5], inhibition of protein synthesis due to tRNA damage cannot explain many activities of Onconase [2,6]. Therefore, another postulated Onconase mechanism is that it acts as an intracellular catalyst for the generation of interfering RNAs (RNAi) which could also trigger apoptosis depending upon the microenvironment of the cell [6]. Further information on the structure and therapeutic potential of Onconase is found in several recent reviews [7-9].

Therapeutic treatment of malignant mesothelioma (MM) remains a major challenge. Prior studies have shown that Onconase induces apoptosis in MM cells and that this effect is tumor cell specific [10]. A cooperative effect was observed between small molecule inhibitors of phosphatidylinositol 3-kinase (PI3K) and Onconase in the killing of MM cells. In MM cells with increased PI3K activity, Rosiglitazone acted cooperatively with Onconase to inhibit cell proliferation [11]. Additional studies are needed to understand the action of Onconase in MM cells. These studies may lead to new combinatorial strategies that may enhance the responsiveness of MM cells to treatment, given that in other tumor types Onconase was shown to be strongly synergistic when combined with other antitumor agents.

For example, the reduction of NF-κB expression in lymphocytic leukemia cells by Onconase appeared to be associated with growth suppression, suggesting that NF-κB and its turnover are important determinants in the anti-proliferative/apoptotic effects of Onconase in this tumor cell context [12]. Also, a positive drug interaction was shown between Onconase and Cepharanthine cytotoxicity when used in combination on various tumor cell lines, and it was postulated that increased cytotoxicity may be associated with Onconase activity in targeting microRNAs and/or NF-κB [13].

Preclinical work coupled with a novel mechanism led to Phase I and Phase I/II clinical trials of Onconase as a single therapeutic agent in patients with a variety of solid tumors [14-16]. Currently, investigations have progressed to confirmatory Phase IIIb clinical trials for the treatment of unresectable MM, one as a single agent [17] and another in combination with doxorubicin. The latter study included subjects with pleural and/or peritoneal MM who had failed one prior systemic therapy, and it was conducted at 27 centers in the U.S. and 31 centers outside the U.S. [18]. Although now closed to enrolment, the FDA approval of premetrexed (Alimta) + cisplatin as front-line treatment had an impact on the type of subjects accrued to this trial and thus included a sizeable population of subjects with previous chemotherapy failure (total, N = 130; Onconase + doxorubicin, N = 65; doxorubicin, N = 65). Hence, the statistical plan provided for an analysis of outcomes based on prior chemotherapy failure, and using this pre-specified comparison, subjects showed a clinically meaningful and statistically significant difference in overall survival favouring the combination of Onconase + Doxorubicin (hazard ratio 1.45; p = 0.033). The survival difference was robust and the statistical significant effect was maintained in a stratified Cox model (hazard ratio 1.548; p = 0.027) [18].

Since Onconase is in advanced clinical development, it is even more important to understand the underlying biological phenomenon connected with its mode of action. Expression profiling is a useful approach to identify pathways altered upon exposure to drugs. Here, a global transcriptional analysis using expression arrays was used to simultaneously search thousands of genes to delineate those that are differentially regulated by Onconase in MM cells. We have identified a subset of genes likely to be generally important in the response of MM cells to Onconase, since they were common to cell lines derived from both epithelial and biphasic tumors. Elucidation of individual genes and their association with signaling pathways should facilitate the development of therapeutic strategies to improve the clinical performance of Onconase.

## Methods

### Cell Lines, Reagents and Treatment

Human MM cell lines M25, M29, M35, M42 and M49 (kindly provided by S.C. Jhanwar [19]) were from pleural tumors. Established cell lines (>passage 10) were propagated in 10 cm dishes (Falcon) in RPMI medium containing 10% FBS. M25 and M35 exhibited biphasic characteristics, whereas M29, M42 and M49 were epithelial in origin. Lyophilized Onconase, from Alfacell Corporation, was dissolved in sterile distilled, deionized water and prepared in medium (RPMI + 10% FBS).

### Cell Viability Assay

An MTS assay (Promega CellTiter 96-AQueous One Solution Assay) was used to analyze the effect of Onconase on cell viability. Cells were cultured overnight in 96-well plates (~1 × 10^4 ^cells/well). Cell viability was assessed after the addition of Onconase at the indicated concentrations for 24, 48 or 72 h. The number of viable cells was assessed by determination of the A_490 nm _of the dissolved formazan product after addition of MTS for 1 h as described by the manufacturer (Promega).

### Expression Array Analysis

**Figure 1:**
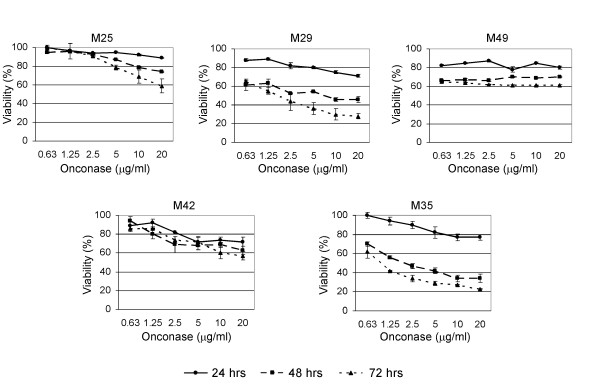
**Human MM cells exhibit different sensitivities to Onconase**. M25, M29, M35, M42, and M49 cells were plated at ~50% confluence, treated with the indicated concentrations of Onconase and evaluated for cell viability by MTS assay at 0, 24, 48 and 72 h following treatment. Graphs represent percent viability of Onconase-treated cells compared to corresponding untreated cells. Bars depict standard error among replicate samples within a representative experiment. Results were validated in repeated experiments.

At ~50 to 80% confluence, all MM cell lines were incubated with Onconase (5 μg/ml) for 24 h in the presence of growth medium containing 10% fetal bovine serum. The concentration and duration of the Onconase treatment for the expression array were selected based on our MTS results (Figure 1), in which there was ≤20% loss of cell viability across most MM cell lines. Cells were harvested, and total RNA was isolated with Trizol, according to the manufacturer's instructions (Invitrogen). Only RNA isolates that had an acceptable concentration and absorbance 260/280 nm ratio from M25, M29 and M49 cells were used.

Expression profiles were generated using Affymetrix Human Genome U133 plus 2.0 Gene Chips (HG U133 plus 2.0) according to the Affymetrix Eukaryote One-Cycle Target Labelling Assay. Briefly, 5 μg of total RNA was used to synthesize double-stranded cDNA, which was *in vitro *transcribed into biotinylated cRNA. The biotinylated cRNA was fragmented and hybridized to GeneChips for 16 hours at 45°C in an Affymetrix Hybridization Oven 640. Arrays were then washed and stained on an Affymetrix Fluidics Station 450 using Affymetrix fluidics protocol EukGE-WS2v5, and subsequently scanned on an Affymetrix GeneChip Scanner 3000 to obtain fluorescence intensities. Relative expression values were generated for each transcript using the Affymetrix MAS5.0 algorithm in GeneChip^® ^Operating Software (GCOS) Version 1.4 with All Probe Sets Scaling of 500. Data have been deposited in NCBI's Gene Expression Omnibus repository http://www.ncbi.nih.gov/geo/ and are available under the accession number "GSE17009".

### Quantitative Real-Time PCR

M49 cells exhibited poor growth characteristics when thawed from multiple frozen isolates and were not used for further analysis. M25, M29, M35 and M42 were used for quantitative real-time PCR. Following treatment with Onconase, both adherent and floating cells were collected and used for RNA isolation. Reverse transcription of RNA to cDNA was performed using a High Capacity cDNA Archive Kit (Applied Biosystems) according to the manufacturer's instructions. Reactions for quantitative real-time PCR were prepared in a 25 μl total volume with TaqMan^® ^Universal PCR MasterMix (Applied Biosystems), TaqMan^® ^Gene Expression Assays (Applied Biosystems) for PTGS2 (COX-2; assay ID: Hs00153133_m1), IL-6 (assay ID: Hs00174131_m1), IL-24 (assay ID: Hs01114274_m1), ATF3 (assay ID: Hs00231069_m1), PTOV1 (assay ID: Hs00363189_g1) or beta-2 microglobulin (assay ID: Hs99999907_m1) and 10-20 ng cDNA per the manufacturer's specifications. Thermal cycling and detection of amplification products were carried out on an ABI PRISM ^® ^7900 Sequence Detection System (Applied Biosystems).

The average C_T _(threshold cycle) values for the target genes and for beta-2 microglobulin were calculated from the replicate values generated for each sample. The average C_T _value for the housekeeping gene, beta-2 microglobulin, was subtracted from the average C_T _value for each target gene in order to normalize values for the amount of total cDNA added to the reaction. Relative quantitation (i.e., fold-difference in expression levels) between untreated and treated samples was calculated using the Ct method (Applied Biosystems User Bulletin Number 2).

### DNA Fragmentation Assay

MM cells were treated with 5 μg/ml Onconase for 24, 48 and 72 h. Adherent and floating cells were collected from untreated or treated cell cultures. Cells were lysed and processed for DNA fragmentation using a Cell Death Detection ELISA Kit (Roche Diagnostics) as per the manufacturer's instructions.

### Gene Ontology Analysis

Gene names were entered into the GeneInfoVis program [20]: http://genenet2.utmem.edu/geneinfoviz/search.php. The resulting matching categories were separated according to the Gene Ontology groupings [21] based on the three broad ontology categories, cellular component [C], molecular function [F] or biological process [P] and then counted. Onto-Express (OE, [22]) was also used to identify those biological processes that were impacted significantly by the Onconase regulated genes (ORGs). Statistical significance values were calculated for each category using either a hypergeometric or binomial distribution depending on the number of genes on the array, as described elsewhere [22].

### Assignment of Genes to Known Pathways

Two principal databases that assign proteins to pathways, KEGG [23] and BioCarta http://www.biocarta.com were accessed through the Cancer Genome Anatomy Project http://cgap.nci.nih.gov/Pathways/Pathway_Searcher. The KEGG and BioCarta resources provide a reference knowledge base for linking genomes to biological systems. The network representation of linkages between ORGs and published pathways was constructed and drawn using the program Cytoscape-2.6.0 available online at http://www.cytoscape.org.

## Results

### Onconase inhibits MM cell viability

Similar to previous studies [10], cell viability assays showed that some MM cell lines are more responsive to Onconase than others. As expected, response was dose and time dependent, with the greatest effect on cell viability observed after 48 to 72 h (Figure 1). None of the Onconase-treated cells reached an IC50 dose response at the 24 h time point. As an unbiased approach to examine gene expression in MM cells in response to Onconase prior to a significant loss of viability, we choose to analyze the 24 h time point across a panel of cell lines that exhibited variable response to Onconase. Subsequent studies validated that a subset of gene expression changes were up-regulated in response to Onconase in MM cell lines.

### Onconase Regulated Genes (ORGs) common to three MM cell lines detected by microarray-based expression analysis

There is precedent for the use of microarrays to identify specific genes that may be important in therapeutic response of MM cells [24,25]. Here, the underlying molecular mechanisms of Onconase-induced loss of MM cell viability were investigated using expression array technology. To select against individual cell line-specific responses, we analyzed only ORGs common to three different MM cell lines. A comparison of the expression profiles in the Onconase-treated and untreated cells revealed 155 genes that were similarly up- of down-regulated in all three cell lines. Most (66%) of the genes were up-regulated (average increase from untreated cells ranged from 1.2- to 14.6-fold), whereas 34% were down-regulated (average decrease compared to untreated cells ranging from 1.3- to 2.5-fold); this suggests that the primary effect of Onconase is activation of gene expression. Many (52) of the genes were associated with one or more cellular pathways. Potential ORGs common to the three cell lines that increased an average of 3-fold or more after Onconase treatment are shown in Table 1. Some of these genes are known to significantly affect apoptosis (IL-24 and TNFAIP3 p = 0.001), transcription (ATF3, DDIT3, MAFF, HDAC9 and SNAPC1, p = 0.009) and inflammation and the immune response (IL-6, p = 0.01 and COX-2, p = 0.001). Although none of the down-regulated genes averaged a 3-fold decrease, PTOV1 was decreased nearly 2-fold.

**Table 1:** ORGs Up-regulated 3-fold or Greater

**Probe Set**^**1**^	**Name**^**1**^	**Change**^**1**^	**Gene Description**^**1**^	**Biological Process**^2^
1560019_at	DLGAP1	14.6	discs large (Drosophila) homolog-associated protein 1	cell-cell signaling
205749_at	CYP1A1	10.8	cytochrome p 450 family 1	electron transport
				drug metabolism
202672_s_at	ATF3	9.4	activating transcription factor 3	transcription
209774_x_at	CXCL2	8.6	chemokine (C-X-C motif) ligand 2	chemotaxis
206569_at	IL-24	6.8	interleukin 24	apoptosis
205207_at	IL-6	6.1	interleukin 6	acute phase response
				B cell differentiation
				cell cell signaling
				cell surface linked signal transduction
				humoral immune response
	TNFAIP3	4.2	tumor necrosis factor, alpha induced protein 3	anti-apoptosis
				apoptosis
				negative regulation of transcription, DNA dependent
204748_at	PTGS2 (COX 2)	4.1	prostoglandin G/H synthase 2	cyclooxygenase activity
				regulation of inflammatory response
209383_at	DDIT3 (GADD153)	3.7	DNA-damage inducible transcripts 3	cell cycle arrest
				transcription
				regulation of transcription, DNA dependent
				response to DNA damage stimulus
367_11	MAFF	3.5	v-maf	parturition
				regulation of transcription, DNA dependent
				transcription from RNA polymerase II promoter
				transcription
205659_ at	HDAC9	3.2	histone deacetylase 9	B cell differentiation
				histone deacetylation
				inflammatory response
				transcription
				regulation of transcription, DNA dependent
202149_at	NEDD9	3.0	neural precursor cell expressed developmentally	actin filament bundle formation
				cytoskeleton organization and biogenesis
205443_at	SNAPC1	3.0	small nuclear RNA activating complex, polypeptide 1, 43 kDa	regulation of transcription, DNA dependent
				transcription from RNA polymerase II promoter
				transcription from RNA polymerase III promoter
				transcription

^1^Affymetrix Data

^2^GeneInfoVis program [20,22]

### Quantitative real-time RT-PCR analysis of gene expression

**Figure 2:**
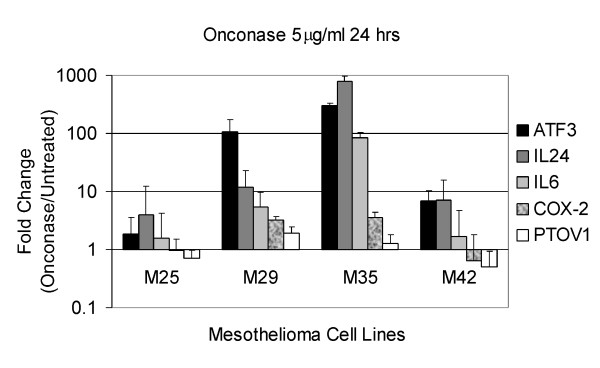
**Quantitative gene expression changes in four human MM cell lines**. RT-PCR was performed as described in the Materials and Methods. Error bars represent standard deviation in the expression level of ATF3, IL-24, IL-6, COX-2 and PTOV1 across three independent experiments in MM cells treated with 5 μg/ml Onconase for 24 h.

Messenger RNA expression of four up-regulated genes and one down-regulated gene were examined by quantitative real-time RT-PCR in two of the three MM cell lines used for the microarray analysis (M25 and M29) plus two additional MM cell lines (M35 and M42) (Figure 2). All four cell lines were treated under conditions identical to that used for the microarray analysis. ATF3, IL-24 and IL-6 were among the most highly induced genes detected in the array analysis (Table 1) and were confirmed to be up-regulated by RT-PCR analysis in all MM cell lines tested. COX-2, another up-regulated gene, was validated to be increased in two of four MM cell lines (Figure 2). PTOV1 expression was slightly decreased or unchanged in the four cell lines. Quantitative gene expression changes demonstrated a wide variation among the four individual cell lines but generally were in the same direction as in the array analysis. ATF3 expression increased from 3-fold to more than 400-fold, IL-24 from 2- to nearly 1000-fold, and IL-6 from 2- to nearly 100-fold. In comparison, COX-2 expression increased ~5 -fold in two cell lines and was unchanged or decreased approximately 2-fold in one line (Figure 2).

**Figure 3:**
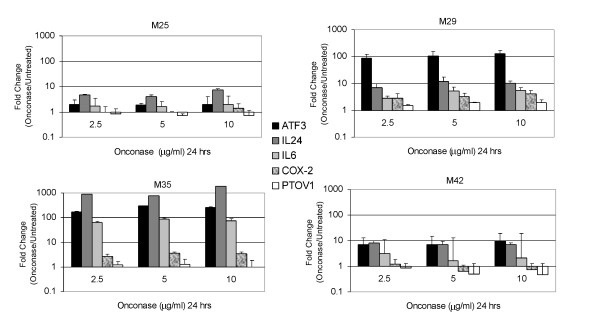
**Expanded series of quantitative RT-PCRs in MM cells treated with different concentrations of Onconase for 24 h**. Human MM cell lines M25, M29, M35, and M42 cells were treated with 0, 2.5, 5 and 10 μg/ml Onconase for 24 h, and RNA was isolated for real-time RT-PCR. Error bars represent standard deviation in the expression level of ATF3, IL-24, IL6, COX-2 and PTOV1 using different dilutions of total RNA. Results were validated in repeated experiments.

**Figure 4:**
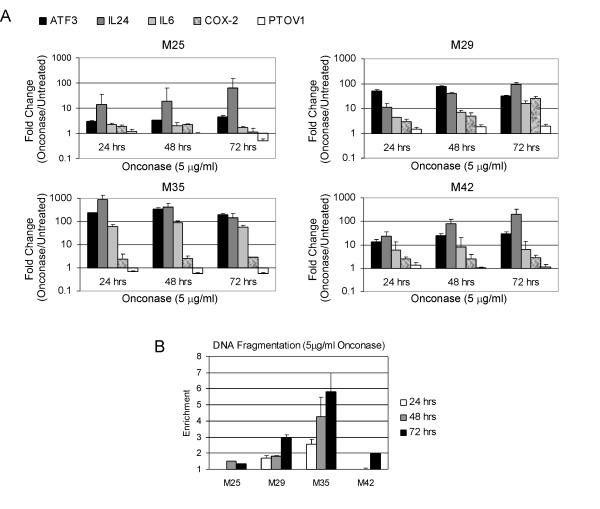
**Time course of expression changes and apoptosis induction in MM cells treated with 5 μg/ml Onconase**. **A**. Bar graph representative of quantitative RT-PCR expression changes. Human MM cell lines M25, M29, M35, and M42 cells were plated at ~50% confluence, treated with 5 μg/ml Onconase for 0, 24, 48 and 72 h, and RNA was isolated. Error bars represent standard deviation in the expression level of ATF3, IL-24, IL-6, COX-2 and PTOV1 using different concentrations of starting RNA. Results were validated in repeated experiments. Increased levels of ATF3 and IL-24 were consistently observed at all time points. **B**. DNA fragmentation analysis of MM cells treated in parallel with samples used for quantitative RT-PCR in "A". M29 and M35 were most sensitive to apoptosis induced by 5 μg/ml Onconase at 48 or 72 h time points. Enrichment of DNA fragmentation in Onconase-treated cells was determined by comparisons with untreated cells as per manufacturer's instructions. Error bars depict standard deviation between replicate fragmentation assays.

We also performed an expanded analysis of gene expression in MM cells treated with Onconase at various doses (2.5 to 10 μg/ml), which showed highly reproducible changes (Figure 3). Moreover, RT-PCR performed on MM cell lines treated for varying times (24 to 72 h) with 5 μg/ml Onconase showed remarkably consistent up-regulated expression of IL-24 as well as of ATF3 and IL-6 (Figure 4A). Among the five genes tested, the gene encoding the pro-apoptotic factor IL-24 is noteworthy in that its expression increased with time in three of four cell lines. Interestingly, induction of ATF3 and IL-24 was highest in M35, a highly responsive MM cell line, and lowest in M25, a poorly responsive cell line, as defined by DNA fragmentation analysis (Figure 4B).

### ORG ontologies

Although investigating individual gene expression can provide insights about important aspects of Onconase action, the overall mechanism is a heterogeneous phenomenon that should also be defined by means of biological processes and pathways. The entire group of ORGs common to three MM cell lines was searched to identify genes that could be characterized for a known ontology. The resulting 155 gene name matches (hits) were used to extract the associated ontologies based on three broad ontology categories: the particular part of the cell (cellular component); the activities of individual gene products (molecular function); and their integration with other genes to form a defined function (biological process). Since each ORG could be associated with one or more of these categories, the number of hits is listed and not the number of genes (Additional file 1). The major biological process ontologies (30 or more hits) were related to cell signaling and signal transduction (34 hits), molecular events involving DNA (34 hits), and transcription (60 hits). The individual function of the ORGs favored metal ion binding and transcription and were mainly associated with membranes and the nucleus (Additional file 1). Other significant biological processes (15 or more hits) involved apoptosis, cell cycle, differentiation/development, cell proliferation/growth, processes involving RNA, metabolism/catabolism, transport and protein molecular events such as phosphorylation, assembly, binding and folding. Taken together, these results (Additional file 1) show that ORGs participate in important cellular events, mostly at the level of transcription.

### Signaling pathways affected by ORGs in MM cell lines

ORG expression was mapped to known metabolic and regulatory pathways using two principal databases, KEGG and BioCarta. To assign genes from the over- or under-expressed gene probes, 155 usable names were generated after removing duplicates and probes that did not correspond to any known genes. Among the 155 ORGs, there were 52 hits mapped to KEGG and/or BioCarta Pathways. The data clearly show clusters of genes in various pathways. Cell cycle and MAPK-associated genes are two examples. Some of the genes (*DUSP6, FAS, HDAC9, IL-6, JAK1, JUN, PLCB4*) are linked to 10 or more known pathways while others such as (*CRY1, DIC1, GTF2H1, JAG1, MAFF, NPPB, PBEF1, PCLO, QPRT, RB1CC1, SAT, SIC2A3, SULT1A3, SUOX, TALDO1*) are only mapped to one. Overall, the data show that after 24 h of treatment, Onconase affects many of the genes involved in classical pathways that control cell growth and death. Some genes, for example *CRY1*, mapped to unusual pathways such as circadian rhythm. While the importance of this pathway to Onconase mechanism of action is unknown, further research may link CRY1 to other more obviously relevant pathways.

**Figure 5:**
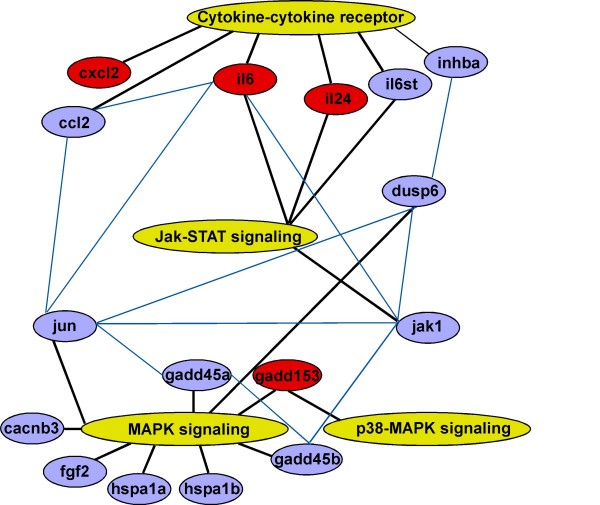
**Network of ORGs and signaling pathways in MM cells**. Major pathways interacting with ORGs (yellow ovals); select ORGs, connected to at least two other genes or pathways (blue ovals and lines); select pathway-associated ORGs expressed at least 3-fold over controls (red ovals). Links between ORGs and major pathways (black lines).

Genes involved in MAPK signaling (p = 0.00006) and cytokine-cytokine-receptor interaction (p = 0.006) were very significantly impacted by Onconase. The ORGs involved are listed in Table 2 and depicted in Figure 5. Jak-STAT (p = 0.06) signaling ORGs are also shown in Table 2 and Figure 5. The p values of the other possible pathway gene clusters ranged from p = 0.2 (Wnt signaling) to p = 0.4 (cell cycle) to p = 0.9 (regulation of actin cytoskeleton).

**Table 2:** ORG Signaling Pathways in MM Cells

MAPK Signaling **p = 0.00006**^**3**^	Cytokine-Cytokine Receptor Interaction p = 0.006	Jak-STAT Signaling p = 0.06
^1^HSPA1A, ^2^heat shock 70 KD	IL-6, interleukin 6	IL-6, interleukin 6
protein A	IL-6ST, interleukin 6 signal	IL-6ST, interleukin 6
HSPA1B, heat shock 70 KD	transducer	signal transducer
protein B	CCL2, chemokine (C-C motif) 2	JAK1, janus kinase 1
CACNB3, Ca channel, voltage	INHBA, inhibin	IL-24, interleukin 24
dependent B3	IL-24, interleukin 24	
FGF2, fibroblast growth factor	CXCL2, chemokine (C-X-C motif) 2	
DUSP6, dual specificity phosphatase 6		
JUN, v-jun sarcoma virus homolog		
GADD45A, growth arrest DNA damage inducible A		
GADD45B, growth arrest DNA damage inducible B		
GADD153, DNA damage inducible transcript 3		

^1^Gene symbol, Affymetrix data

^2^Gene description, Affymetrix data

^3^p values from [22]

### Interaction of ORGs in MM cell lines

In addition to Figure 5, Table 3 further illustrates the interconnected relationship of ORGs and known pathways. More than half of the 35 shared pathways involve signaling. One cluster (data not shown) contained CYP1A1 and COX-2, two of the more highly up-regulated ORGs (Table 1). CYP1A1 (tryptophan, linoleic and fatty acid metabolism pathways) is connected via the arachidonic acid metabolism pathway to COX-2 (eicosanoid metabolism pathway). The ORGs in the cytokine-cytokine receptor and Jak-STAT pathways are directly linked through shared genes, e.g. IL-6, IL-6st and IL-24. Other relevant linkages are through second or third order shared genes and pathways. For example, MAPK to DUSP6 is linked further to the cytokine-cytokine receptor pathways via TGFβ signaling (Figure 5) and INHBA. Numerous other linkages can also be seen. Thus, Onconase-induced signals are propagated through the cellular membrane and intracellular milieu. This implies that Onconase initiates a cascade of ORG-mediated responses leading to growth inhibition and apoptosis in human MM cell lines.

**Table 3:** Shared pathways

1	(LDL) pathway during atherogenesis ^1^	19	IL-2 signaling
2	ATM signaling	20	IL-10 anti-inflammatory signaling
3	Axon guidance	21	IL-2 receptor beta chain T cell activation
4	B cell receptor signaling	22	Keratinocyte differentiation
5	Calcium signaling	23	Leukocyte transendothelial migration
6	Cell adhesion molecules (CAMs)	24	Long-term depression
7	Cell cycle	25	Long-term potentiation
8	Colorectal cancer	26	Natural killer cell cytotoxicity
9	Complement and coagulation cascades	27	Oxidative stress via Nrf2
10	Control by vitamin D receptor	28	PDGF signaling
11	EGF signaling	29	Pertussis toxin-insensitive CCR5 signaling Macrophage
12	Epithelial cell signaling H. pylori infection	30	T cell receptor signaling
13	FAS signaling (CD95)	31	TGF-beta signaling
14	Fc epsilon RI signaling	32	Tight junction
15	Focal adhesion	33	Toll-like receptor signaling
16	Gap junction	34	VEGF signaling
17	GnRH signaling	35	Wnt signaling
18	Hematopoietic cell lineage		

^1 ^Pathway description

## Discussion

Microarray technology is a powerful tool for investigating cellular responses to drugs because it monitors thousands of genes simultaneously. The present study is the first to use microarray-derived transcriptional profiling to identify ORGs common to multiple human MM cell lines. Many ORGs are associated with cellular signal transduction, proliferation and differentiation. Transcription and DNA processes connected mainly with membranes and the nucleus were the most affected ontologies. All of this is consistent with the reprogramming of cellular regulatory systems by Onconase. Important new insights into Onconase mechanism include the convergence of cellular responses to RNA damaging and standard DNA-damaging drugs, and ORG interaction with genes involved in MAPK signaling.

IL-24 was originally identified as *mda*-7, a gene selectively up-regulated during terminal differentiation of melanoma cells [26] and later classified as a member of the IL-10 family [27]. Recently, an adenovirus encoding the IL-24 gene has been shown to have selective anticancer effects in cell culture and in animal models (reviewed in [28]). IL-24 also sensitizes cells to radiation and other conventional therapies, inhibits angiogenesis and exhibits bystander effects in animal models. A replication-incompetent adenovirus encoding IL-24 (Ad. *mda*-7) has undergone evaluation in a Phase I clinical trial and showed rapid clinical advancement [28]. However, gene therapy protocols exhibit limitations associated with local delivery and adverse immune reactions. The specific high level induction of IL-24 by NSAIDs has generated enthusiasm for increasing systemic levels of IL-24 that might be effective without the limitations of adenoviral delivery [29]. The present work shows that Onconase also induces IL-24 mRNA in MM cells. This finding has implications for understanding the basis for the selective antitumor effects of Onconase in cell culture experiments [30] and its well tolerated activity in patients [14]. Up-regulation of IL-24 might also be related to the enhanced radiation sensitivity of non-small cell lung cancer (NSCLC) cells treated with Onconase [31], because IL-24 has been shown to sensitize NSCLC xenograft tumors to radiation [32].

In view of these findings, combination therapy with Onconase and NSAIDs should be considered in treating MM patients if preclinical efficacy and safety studies warrant it. NSAIDs decrease the expression of COX-2 and genes involved in the formation of inflammatory prostaglandins from polyunsaturated fatty acids such as arachidonic acid [33] in MM cells [34]. Thus, the use of NSAIDS could moderate any potential effects of Onconase on COX-2 expression, while the combined effect of both agents might induce more IL-24 than either agent alone. Indeed, an NSAID has been shown to increase ectopic expression of IL-24 that augmented adenovirus delivery leading to the proposed use of more than one inducer of IL-24 to achieve superphysiological levels of this pro-apoptotic factor in tumors [35].

ATF3 has not previously been associated with Onconase activity. ATF3 (activating transcription factor 3), a member of the ATF/CREB family of transcription factors, participates in cellular processes to adapt to extra/intracellular changes [36]. It is expressed at low levels in normal and quiescent cells but can be induced by a variety of extracellular agents, including radiation [37] and other genotoxic stress signals [38]. The consequences of ATF3 induction result in tumor growth inhibition and suppression of Ras-stimulated tumorigenesis [37,38]. ATF3 mRNA was up-regulated in MM cell lines used in the microarray expression analysis as well as in two additional human MM cell lines examined, indicating that the Onconase up-regulation of ATF3 is not cell line specific.

Chemotherapeutic agents that damage DNA also induce ATF3. Camptothecin and etoposide, DNA topoisomerase inhibitors [39], as well as the anthracycline antibiotic doxorubicin [40] have all been shown to induce ATF3 in tumor cells. Stably or transiently transfected ATF3 accelerated drug-induced apoptosis [39]. 5-Fluorouracil (5-FU) is used in the treatment of gastrointestinal, breast, and head and neck cancers [41]. Treatment of colon cancer cell lines with 5-FU induced ATF3 along with other DNA damage response genes (GADD34, GADD45α, PCNA) [42].

Overexpression of more than one GADD gene causes synergistic growth inhibition and/or apoptosis [43]. Therefore the combined induction of GADD45α, GADD45β and GADD153 by Onconase seen in our microarray analysis suggests these genes may act synergistically to inhibit MM cell viability.

ORGs very significantly affect MAPK signaling in MM cells. This is consistent with MAPK regulation of ATF3 [36] as well as the GADD genes [44,45]. Sequential activation of protein kinases within the MAPK pathways is a common mechanism of signal transduction in many cellular processes including the stress response, and apoptosis [46]. In fact, MAPK signaling is common to the action of many divergent chemotherapeutic agents (reviewed in [47]). In addition to MAPK, AKT signaling has been shown to influence tumor cell responsiveness to chemotherapeutics. Although not addressed in this study, a previous report noted that Onconase induces apoptosis in MM cells, and that MM cells with the highest AKT activation were more resistant to the drug [10].

Recently, genotoxic stress and apoptosis were also associated with RNA genes [48]. MicroRNAs (miRNAs), products of non-coding RNA genes, that are transcribed by RNA polymerase II, are processed and exported to the cytoplasm where they bind to the 3' UTRs of target mRNAs to inhibit translation [49]. Numerous miRNAs are deregulated in human cancers, consequently affecting their target mRNAs and subsequent protein products [49]. Onconase is known to damage non-coding RNA (i.e., tRNA) to inhibit protein synthesis, but its effect on apoptosis has been attributed, at least partly, to other unknown cellular targets [2], possibly miRNAs [6]. Interestingly, nine ORGs affect transcription from the RNA polymerase II promoter, and one of these, CEBPB, is a target of miRNA-155 [50]. Moreover, miRNA-155 is up-regulated in some cancers [51], and its levels are decreased by Onconase in MM cells (K. Shogen, unpublished data).

## Conclusion

Collectively, the results presented here show that a RNA-damaging drug can impact many of the same important genes and pathways as standard chemotherapeutics. Future studies are needed to address whether ATF3 and IL-24 act as key pro-apoptotic genes to mediate the chemotherapeutic response of MM cells to Onconase. Moreover, it remains to be determined whether up-regulation of IL-6 and COX-2 expression could impact the responsiveness of MM patients to Onconase. Targeting these pro-inflammatory proteins may lead to improvements in Onconase treatment protocols. Current findings implicate several new genes in the response of MM cells to Onconase, and further studies are expected to elucidate whether up-regulation of these genes may serve as new biomarkers of response to Onconase.

## Abbreviations

C_T_: threshold cycle; MM: malignant mesothelioma; NSAIDs: nonsteroidal anti-inflammatory drugs; ORG: Onconase regulated genes; tRNA: transfer RNA.

## Competing interests

The authors declare that they have no competing interests.

## Authors' contributions

DAA, SMR, JRT and KS designed experiments. DAA, JP and MC performed experiments. DAA, SMR, JP, JVM and MC analyzed the data. DAA, SMR, JP and JVM were responsible for data management and preparation of tables and figures. DAA, SMR, JRT and KS wrote the manuscript. All authors read and approved the final manuscript.

## Pre-publication history

The pre-publication history for this paper can be accessed here:

http://www.biomedcentral.com/1471-2407/10/34/prepub

## Supplementary Material

Additional file 1 **Supplemental Table 1**. Table detailing Onconase regulated gene (ORG) ontologies. Click here for file
