# Supplementary material for: Onconase responsive genes in human mesothelioma cells: implications for an RNA damaging therapeutic agent
Source: BMC Cancer. 2010 Feb 5;10:34. doi: 10.1186/1471-2407-10-34 (PMC2829496; doi:10.1186/1471-2407-10-34)
Supplement: Additional file 1 — Supplemental Table 1. Table detailing Onconase regulated gene (ORG) ontologies. [file 1471-2407-10-34-S1.PDF]

Additional files

Additional file 1:

Supplemental Table 1. Table detailing Onconase regulated gene (ORG) ontologies

This file can be viewed with: Microsoft Word

ORG Ontologies

Cellular Component	#Hits[1]	Molecular Function	#Hits	Biological Process	#Hits
cytoplasm	12	ATP/ATPase	14	apoptosis	18
cytoskeleton	5	calcium	11	cell-cell signal transduction	34
extracellular	14	DNA binding	14	cell cycle	15
membrane	40	metal ion binding	23	differentiation/development	23
mitochondria	8	nucleic acid/nucleotide	11	immune response	11
nuclear	41	protein binding	11	inflammatory response	5
		structural activity	5	cell proliferation/growth	19
		transcription	27	chromatin/chromosome	7
		transferase activity	11	transcription	60
				DNA	34
				RNA	17
				metabolism/catabolism	24
				transport	15
				phosphorylation/kinase	11
				protein processes	22

[1](Hits) Each gene could be associated with one or more of these ontologies
